# A Novel Pyroptosis-Related Prognostic Signature for Risk Stratification and Clinical Prognosis in Clear Cell Renal Cell Carcinoma

**DOI:** 10.1155/2022/8093837

**Published:** 2022-03-09

**Authors:** Xiao-qiong Pan, Wen Huang, Ling-wei Jin, Hua-zhen Lin, Xiao-yan Xu

**Affiliations:** ^1^Department of Traditional Chinese Medicine, The Second Affiliated Hospital of Wenzhou Medical University, Wenzhou, Zhejiang, China; ^2^Department of Nephrology, The Second Affiliated Hospital of Wenzhou Medical University, Wenzhou, Zhejiang, China

## Abstract

Emerging research has substantiated that pyroptosis-related biomarkers were mightily related to the clinical outcome of patients with clear cell renal cell carcinoma (ccRCC). However, a single-gene biomarker's moderate predictive power is insufficient, and more accurate prognostic models are urgently needed. We conducted this investigation in order to develop a robust pyroptosis-related gene signature for use in risk stratification and survival prognosis in colorectal cancer. We downloaded transcriptomic data and survival information of ccRCC patients from TCGA. Bioinformatic methods were used to generate a pyroptosis-related gene signature based on data from TCGA training cohort. ROC curve, uni- and multivariate regression analyses were used for the prognostic assays. What is more, we explored the relationship between model-based risk score and the tumor microenvironment. Herein, 11 pyroptosis-related hub genes (CASP9, TUBB6, NFKB1, BNIP3, CAPN1, CD14, PRDM1, BST2, SDHB, TFAM, and GSDMB) were determined as risk signature for risk stratification and prognosis prediction for ccRCC. Kaplan-Meier curves, ROC curves, and risk plots were employed to analyze and verify its performance in all groups. Multivariate assays exhibited that risk score could be an independent prognostic factor for patients' OS. ESTIMATE algorithm showed a higher immune score in the group of high-risk. Overall, a novel pyroptosis-related gene signature generated can be employed for prognosis prediction of ccRCC patients. This may assist in individual treatment of clinical decision-making.

## 1. Introduction

Renal cell carcinoma is a malignant tumor originating from the urinary tubular epithelial system of the renal parenchyma and accounts for 2-3% of malignant cancers in adults [1]. RCC can have several histological subtypes, the most common of which is clear cell renal cell carcinoma (ccRCC) and accounts for 70%-85% of cases [2]. ccRCC often lacks clinical manifestations in its early stages and is detected in more than half of patients during physical examinations or other diseases. Additionally, about 30% of the patients are already found to have distant metastases at the time of initial consultation, often presenting with pathological fractures, cough, hemoptysis, etc.

Before 2005, cytokine therapy was used to treat advanced renal cell carcinoma, but the effect was poor [3]. With targeted drugs and immune checkpoint inhibitors, some patients have improved survival [4]. However, the prognosis of most patients remains unsatisfactory. What is more, some patients are not sensitive to the above treatments and may suffer from side effects, emphasizing individualized treatment for patients with ccRCC [5]. Therefore, an effective prediction model is required for the prediction of the stratification of patients accurately. In recent years, the availability of large public datasets of gene expression provides an opportunity to develop new predictive tools based on prognosis-related genes.

Pyroptosis is a programmed cell death that induced by gasdermin-mediated inflammasomes [6]. Pyroptosis is recognized as a crucial part in removing various bacterial and viral infections [7]. Dysregulation of pyroptosis may lead to lowered efficiency and malfunction of pathogen clearance, motivating impaired adaptive immune defenses and consequently tissue damage [8]. Gasdermins belong to the gasdermin superfamily, including six members in human. GSDMD and GSDME were reported as essential participants in the pyroptosis. In the classic inflammatory pathway, a series of pathogen-associated molecular patterns (PAMP) or danger-associated molecular patterns (DAMP) activate inflammasomes. Then, GSDMD is cleaved into two fragments by the activated caspase-1. Additionally, Rogers et al. demonstrated that GSDME is cleaved explicitly by caspase-3 to produce its N-terminal fragment, which is perforated at the plasma membrane to induce pyroptosis.

Recently, emerging evidence indicated that pyroptosis was chemically induced in tumor cells in the absence of any bacterial or viral infection [9]. In some cases, particularly in cancer treatment, cell death may be favorable for human health [10]. The activation of pyroptosis can inhibit the onset and progression of cancer [11]. Chemotherapy drugs, reagents, and natural products have been shown to activate pyroptosis and inhibit tumor progression. For instance, in lung cancer, topotecan, bleomycin, actinomycin-D, and doxorubicin can induce pyroptosis through caspase-3 cleavage of a gasdermin [12]. In melanoma cells, loss of eEF-2K inhibited autophagy and increased pyroptosis, thereby modulating cellular sensitivity to doxorubicin [13]. Moreover, As2O3 nanoparticles (As2O3-NPs) increase the expression of GSDME-N in hepatocellular carcinoma cells, which resulting in pyroptosis [14]. In osteosarcoma, dioscin suppresses the growth of tumor cells via inducing apoptosis, pyroptosis, and in vitro and in vivo [15].

In the initial and progression of ccRCC, there are limited studies on the pyroptosis-associated genes. During the occurrence and development of ccRCC, the specific mechanisms of pyroptosis remain unclear. To take advantage of the complementary value of genes and clinicopathological features, a complete predictive model for individuals with ccRCC was developed by combining pyroptosis-associated genes with clinical factors.

## 2. Materials and Methods

### 2.1. Data Collection

The transcriptomic data, including 539 ccRCC specimens and 72 nontumor specimens, were downloaded from The Cancer Genome Atlas database (https://portal.gdc.cancer.gov/). All data have been standardized by FPKM. After excluding duplicate samples and samples lacking survival data, we finally obtained RNA sequencing data of 72 normal kidney specimens and 530 ccRCC specimens. The correlated clinical characteristics of all patients were also obtained.

### 2.2. Identification of Differentially Expressed PRGs (DE-PRGs)

The GeneCards database has a thorough list of PRGs, which was used to create this list. For the purpose of identifying DE-PRGs with an adjusted *P* 0.05 or less between ccRCC and nontumorous samples, the Wilcoxon tests and the “limma” R package were employed.

### 2.3. GO and KEGG

A GO and KEGG pathway enrichment analysis was performed on DE-PRGs using the “clusterProfiler” R package to investigate the biological functions and processes of the DE-PRGs under investigation [16].

### 2.4. Construction and Validation of a Prognostic Pyroptosis-Related Gene Signature

All patients were randomized into four groups at random using a 6 : 2 : 1 : 1 ratio: the training group, testing set one, testing set two, and testing set three. Then, we began to construct a risk model. The hub PRGs were screened using multivariate tests, and a model was constructed, and the regression coefficients of hub PGRs were also obtained. Secondly, each ccRCC patient was assigned a risk score based on the following formula: exp gene 1∗*β* gene 1 + exp gene 2∗*β* gene 2 + exp gene 3∗*β* gene 3 + ⋯exp gene n∗*β* gene n (exp gene stands for the relative expressions of hub PGRs). Thirdly, patients with ccRCC patients were classified into low- and high-risk subgroups according to the median risk score. Fourthly, Kaplan-Meier assays were applied to estimate overall survival rates. Finally, we used the “Survival ROC” R software package to evaluate the predictive abilities of our model.

### 2.5. Identification of the Independent Prognostic Factors and Construction of a Nomogram

In the present study, uni- and multivariate assays were applied for the determination of independent prognostic factors. Using the independent prognostic factors, we construct a nomogram for ccRCC patients.

### 2.6. Association of Risk Score and Tumor Microenvironment

In this study, the immune and stromal scores were examined using the ESTIMATE algorithm. Then, CIBERSORT algorithm was used to determine the TIICs' content. We quantitatively compared the distribution of TIIC subtypes between low and high subgroups. *P* < 0.05 was statistically significant. Given that ICIs have been used therapeutically to treat ccRCC, we also investigated if the risk score may be associated with regulators associated with the ICI class.

### 2.7. Cell Culture and Cell Transfection

The ccRCC cell lines (786-O, ACHN, A498) and human renal tubular epithelial cell line (HK-2) were purchased from the American Type Culture Collection (ATCC, USA). DMEM medium (Gibco, USA) with 10% fetal bovine serum (FBS, Gibco) was applied to culture the cells with 5% CO_2_ at 37°C.

786-O and ACHN cells were seeded at 70-80% confluency before transfection and transfected with siRNAs using Lipofectamine 2000 (Invitrogen, Pudong, Shanghai, China) based on Manufacturer's Guide. The effects of BST2 were determined by the use of RT-PCR. All the siRNAs (30 nM) were synthesized by Genepharma (Shanghai, China).

### 2.8. Cell Counting Kit-8 (CCK-8) Assay

The cellular viabilities were examined using CCK-8 (CP002, SAB, Nanjing, Jiangsu, China) based on the manufacturer directory. The absorbance was used to plot proliferation curves at each time point (24, 48, 72, and 96 h).

### 2.9. Statistical Analysis

A gene signature was created by the use of multivariate assays, which confirmed the existence of survival-related genes. The prognostic values of the model were described using Kaplan-Meier assays and a log-rank test. Time-dependent ROC curve was used to examine the predictive abilities of risk model. The Wilcoxon test was used for the comparison of immune infiltrating cells and immunosuppressive molecules between the two groups. Student's *t*-test was performed to analyze the significance of differences between two groups. *P* value < 0.05 was considered statistically significant. All statistical analyses were performed using R (version 3.6.1) and SPSS 20.0 software (Chicago, IL, USA).

## 3. Results

### 3.1. Data Preparation

**Figure 1:**
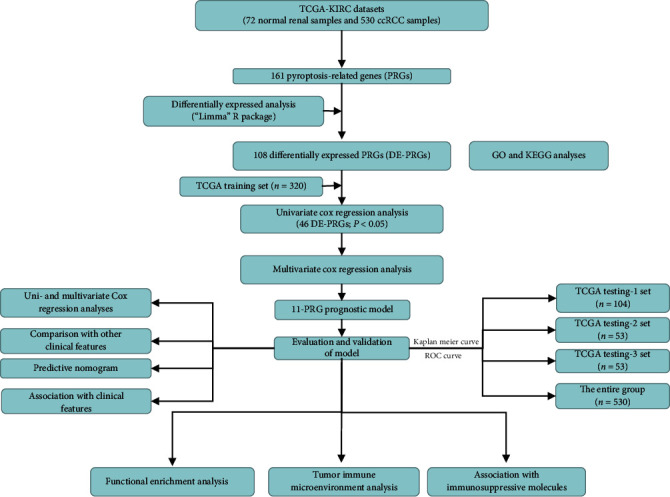
A flowchart depicting the gene signature of ccRCC found in this study, as well as its entire analysis.

The flowchart of this study is shown in Figure 1. 530 ccRCC patients from TCGA datasets were collected for this study.

### 3.2. Identification of DE-PRGs

**Figure 2:**
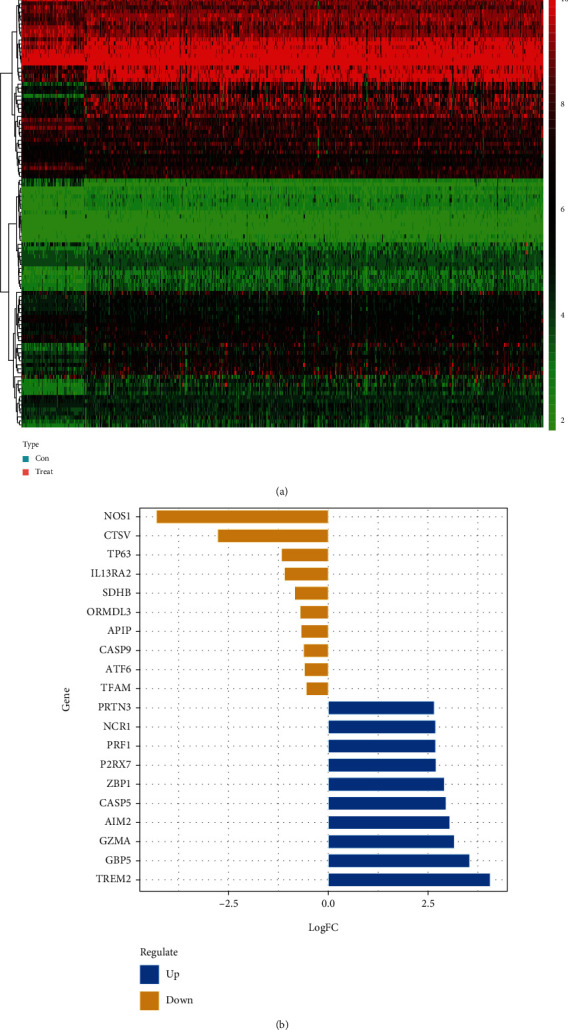
Identification of DE-PRGs between normal specimens and ccRCC specimens. (a) The heat map of DE-PRGs. (b) The top ten upregulated and downregulated PRGs.

DE-PRGs between ccRCC specimens and noncancerous specimens were screened by the use of the “limma” R program. Herein, a total of 108 DE-PRGs (upregulated genes: 84 and downregulated genes: 24 were identified).  Figure 2(a) shows the heat map of all DE-PRGs.  Figure 2(b) depicts the top ten PRGs that were up- and downregulated in ccRCC.

### 3.3. GO and KEGG Analyses of DE-PRGs

**Figure 3:**
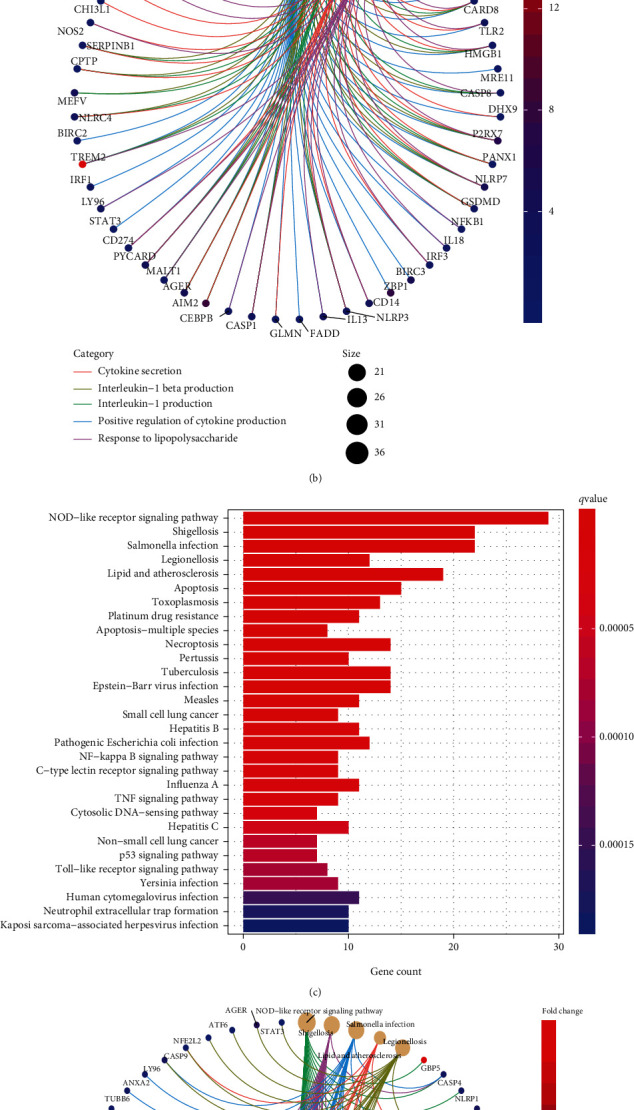
GO and KEGG assays of DE-PRGs. (a) GO analysis of DE-PRGs; (b) enriched GO enrichment terms and corresponding DE-PRGs; (c) KEGG signaling pathway analysis of DE-PRGs; (d) enriched cancer-related pathways and corresponding DE-PRGs.

Using the “clusterProfiler” R package, a functional enrichment analysis of these DE-PRGs was carried out in order to gain a better understanding of the biological systems and potential pathways that these DE-PRGs are involved in. As exhibited in Figure 3(a), in the BP group, DE-PRGs were mainly involved in cytokine secretion and response to lipopolysaccharide. For CC, DE-PRGs were related to vesicle lumen, cytosolic part, membrane region, membrane microdomain, and the membrane raft; moreover, significantly enriched MF included cysteine-type peptidase, endopeptidase activity, and ubiquitin protein ligase binding.  Figure 3(b) depicts the five considerably enriched GO keywords as well as the pertinent DE-PRGs that were involved in their developments. KEGG analysis showed that DE-PRGs are mainly enriched in apoptosis, salmonella infection, Shigellosis, and NOD-like receptor signaling pathway (Figure 3(c)).  Figure 3(d) displays the five significantly enriched signaling pathways.

### 3.4. Construction and Validation of a Prognostic Signature Based on Survival-Related PRGs

**Figure 4:**
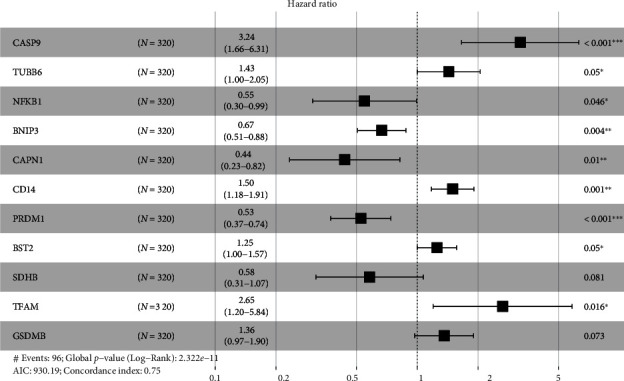
Multivariate Cox regression analysis identified 11 PRGs that were linked with poor prognosis. ^∗^*P* < 0.05, ^∗∗^*P* < 0.01, and ^∗∗∗^*P* < 0.001.

**Figure 5:**
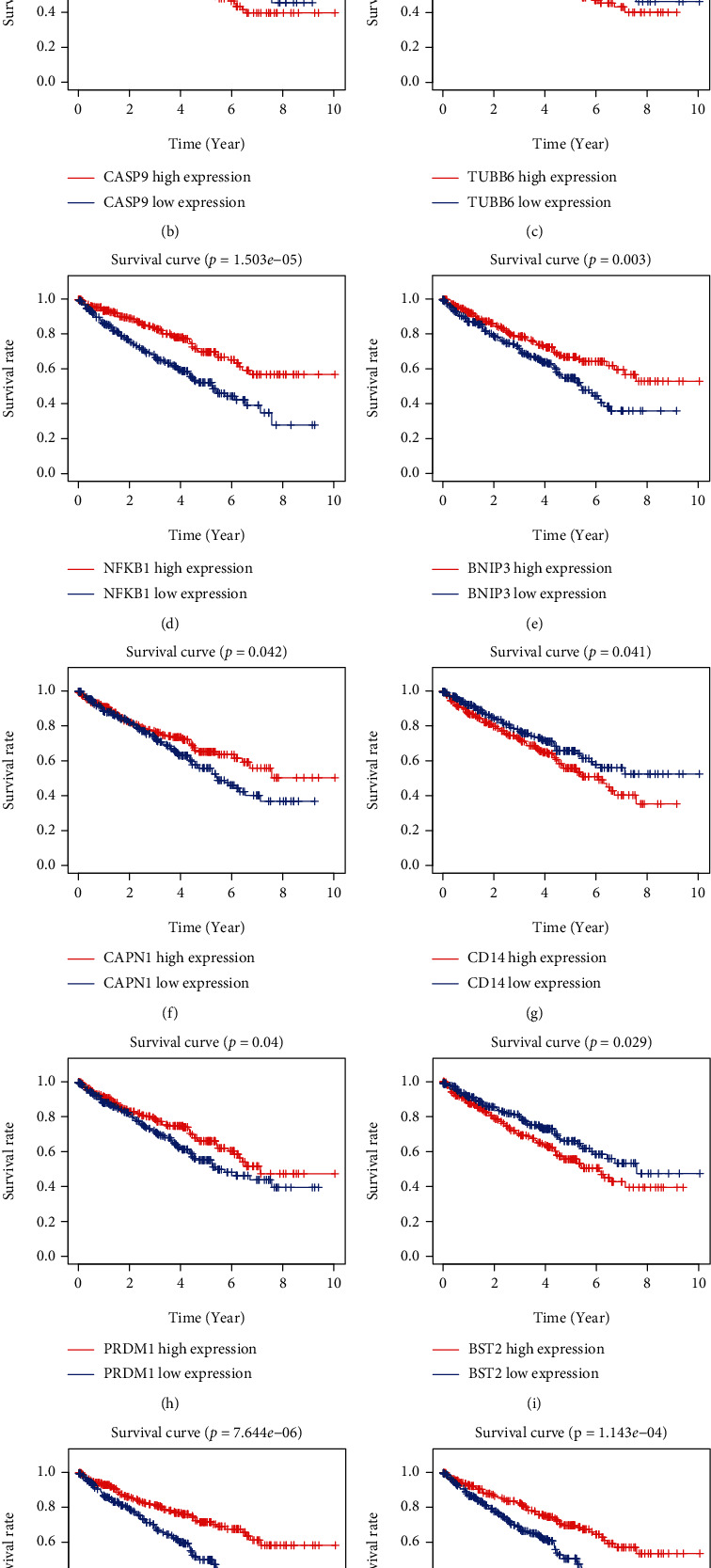
Genetic mutation and survival analysis of hub PRGs. (a) Genetic alteration of hub PRGs in ccRCC patients. (b–l) Kaplan-Meier curves of hub PRGs.

**Figure 6:**
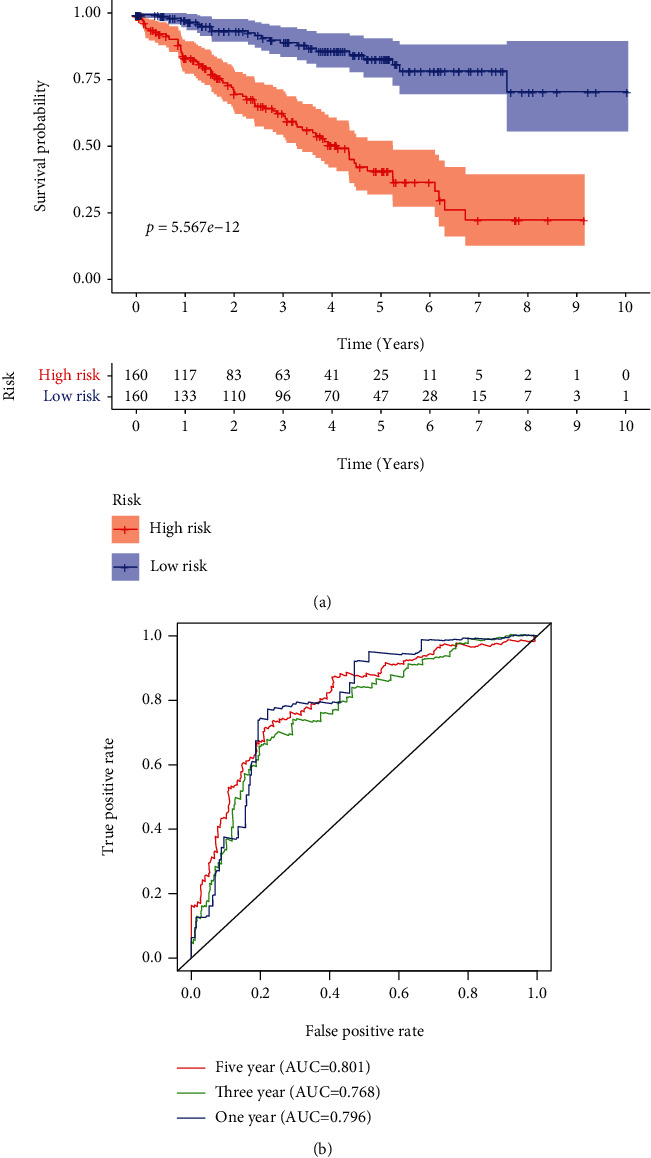
Incorporation of 11 hub PRGs into the development of a predictive signature. (a) In the training cohort, the survival curves for high- and low-risk subgroups were plotted; (b) time-dependent ROC curve.

**Figure 7:**
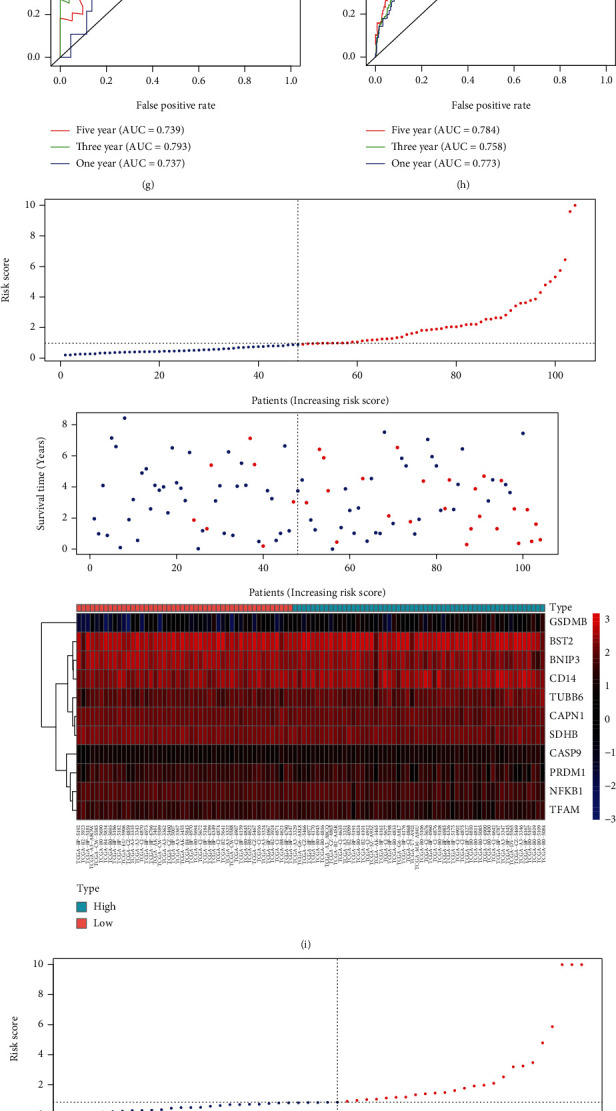
Testing the predictive signature based on 11 pyroptosis-associated genes in different cohorts to determine its reliability. (a–d) The Kaplan-Meier survival curves of the 11-PRG prognostic signature in several groups. (e–h) Time-dependent ROC curves. (i–l) Risk score distribution, survival status, and heat map.

To establish a prognostic signature, univariate assays were conducted to screen survival-associated PRGs, and 46 PRGs remarkably associated with OS of patients with ccRCC in TCGA training set were identified (*P* < 0.05) (Table S1). Subsequently, to create a gene signature, multivariate assays were used in conjunction with each other. Eventually, 11 hub PRGs (CASP9, TUBB6, NFKB1, BNIP3, CAPN1, CD14, PRDM1, BST2, SDHB, TFAM, and GSDMB) were screened (Figure 4). The genetic mutations of 11 hub PRGs are displayed in Figure 5(a). Additionally, Figures 5(b)–5(l) show the Kaplan-Meier curves of 11 hub PRGs in ccRCC. The regression coefficients of 11 hub PRGs are exhibited in Table S2. We calculated the risk score by the use of the following formula: risk score = (1.174∗CASP9) + (0.359∗TUBB6) + (−0.6∗NFKB1) + (−0.404∗BNIP3) + (−0.827∗CAPN1) + (0.404∗CD14) + (−0.643∗PRDM1) + (0.225∗BST2) + (−0.544∗SDHB) + (0.973∗TFAM) + (0.307∗GSDMB). Subsequently, in the training set, when comparing high-risk patients to low-risk patients, Kaplan-Meier assays revealed that high-risk patients had a shorter overall survival time (*P* < 0.001) (Figure 6(a)). ROC assays also demonstrated its diagnostic value (Figure 6(b)). Then, each patient's risk score in all groups was likewise calculated. The Kaplan-Meier assays indicated a distinctly excellent OS in the low-risk group (testing-1: *P* < 0.01, testing-2: *P* < 0.01, testing-3: *P* < 0.05, and entire group: *P* < 0.001) (Figures 7(a)–7(d)). The AUC of the gene signature in the testing-1 cohort for 1-year, 3-year, and 5-year OS were 0.781, 0.746, and 0.770 (Figure 7(e)). The AUC of the gene signature in the testing-2 cohort and testing-3 cohort is exhibited in Figures 7(f) and 7(g). The AUC of the gene signature is shown in Figure 7(h). The distribution of the risk score and survival status and the expression of 11-PRGs in the all groups are presented in Figures 7(i)–7(l), respectively.

### 3.5. Identification of Independent Prognostic Factors, Construction of a Nomogram, and Correlation of Prognostic Signature with Clinical Features

**Figure 8:**
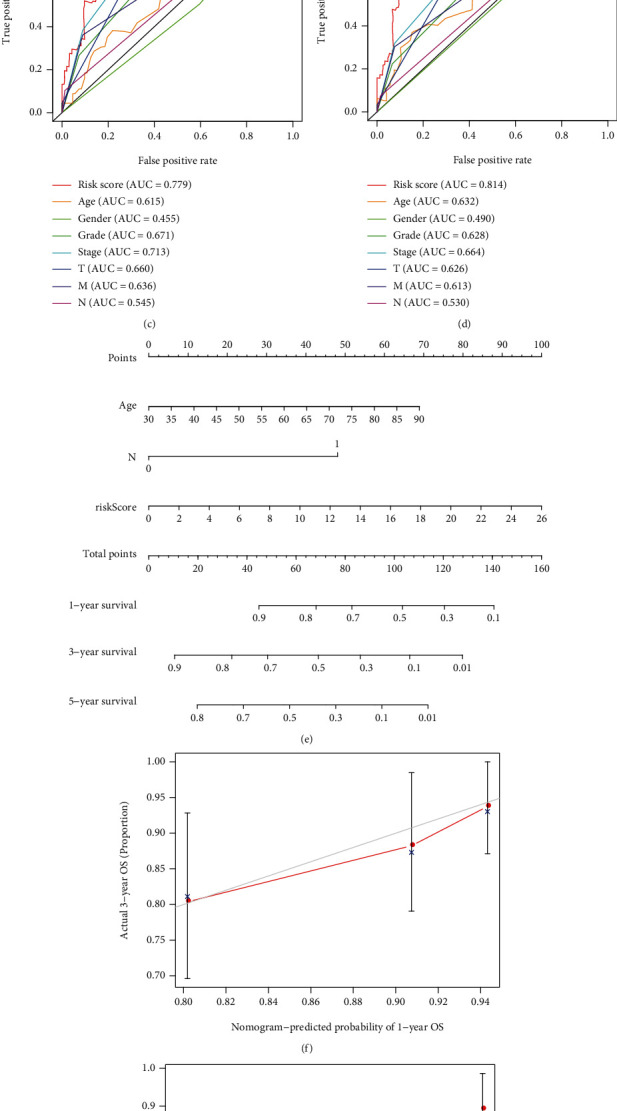
Separate evaluation of prognosis and development of nomograms. (a) Univariate assays for risk score and other clinical factors. (b) Multivariate assays for risk score and other clinical factors. (c, d) A comparison of 3-year and 5-year ROC curves. (e) Nomogram predicting 3- and 5-year overall survival of ccRCC patients. (f–h) Calibration curves.

**Figure 9:**
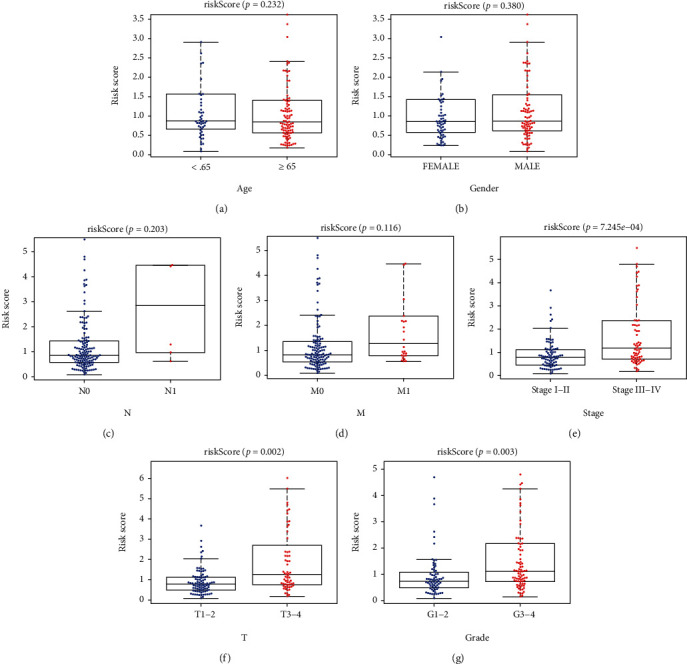
The associations between risk scores and clinical features of ccRCC patients. (a) Age; (b) gender; (c) N status; (d) M status; (E) stage; (F) T status; (G) grade.

Univariate assays revealed that age, histological grade, clinical stage, and risk score were distinctly related to OS of ccRCC patients (Figure 8(a)). Further multivariate assays indicated that age, N stage, and risk score were independent prognostic factors for ccRCC patients (Figure 8(b)). In addition, among other clinical factors, we discovered that risk score had the highest AUC in predicting 3- and 5-year OS in ccRCC (Figures 8(c) and 8(d)). Moreover, we used age, N stage, and risk score to develop a prognostic nomogram (Figure 8(e)); calibrate curves revealed that the nomogram performed well at overall survivals in ccRCC cases (Figures 8(f)–8(h)), suggesting the robust prognostic abilities of this new nomogram. Besides, we assessed the association of risk score with clinical features, and the result indicated that there was no statistical difference between risk score and the above clinical features (Figures 9(a)–9(d)). However, elevated risk score was significantly correlated with advanced clinical stage (*P* < 0.001, Figure 9(e)), T stage (*P* < 0.01, Figure 9(f)), and higher histological grade (*P* < 0.01, Figure 9(g)).

### 3.6. Functional Enrichment Analyses

**Figure 10:**
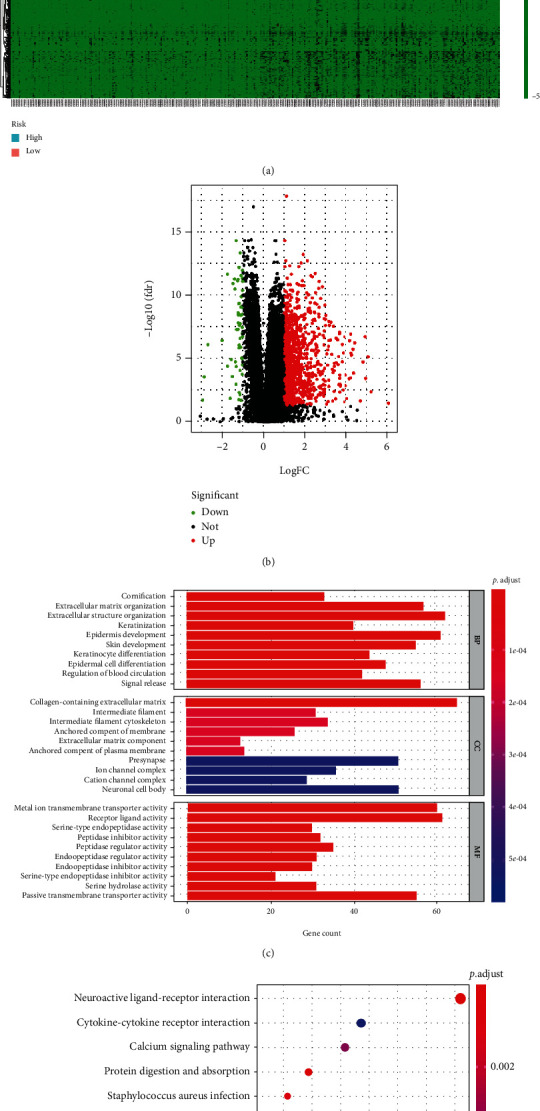
Functional assays of DEGs between low and risk groups. (a) Heat map of DEGs; (b) volcano map of DEGs; (c) GO assays; (d) KEGG assays.

A total of 1,179 DEGs, which met the criteria with the absolute value of logFC > 1 and FDR < 0.05, were identified. Figures 10(a) and 10(b) show the pattern of DEGs, respectively. DEGs were significantly enriched in cornification, extracellular structure organization, keratinization, and epidermis development; in terms of cellular components, DEGs were distinctly involved in intermediate filament cytoskeleton and extracellular matrix component. Moreover, DEGs were noticeably involved in metal ion transmembrane transporter activity, receptor ligand activities, peptidase inhibitor activities, and peptidase regulator activities (Figure 10(c)). KEGG pathway analysis showed that DEGs were mainly enriched in Staphylococcus aureus infection, neuroactive ligand-receptor interaction, coagulation cascades, hypertrophic cardiomyopathy, calcium signaling pathway, pancreatic secretion, and IL-17 signaling pathway (Figure 10(d)).

### 3.7. Tumor Immune Microenvironment Analyses

**Figure 11:**
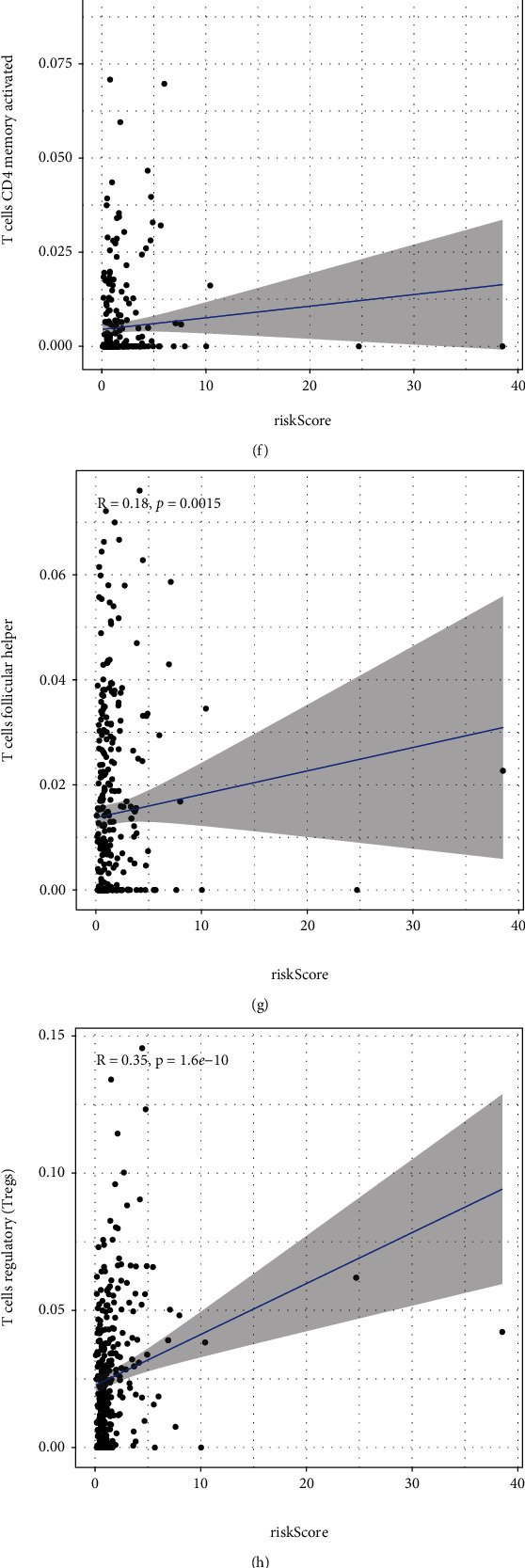
Associations of risk score with tumor immune microenvironment in ccRCC. (a) Differences in immune score between low- and high-risk subgroups. (b) Differences in stromal score between high- and low-risk subgroups. (c) The expressing pattern of immune cells based on risk score. (d–i) Correlation analysis confirmed that risk score was positively associated with immune infiltration levels of immune cells. (j–l) Correlation assays demonstrated that risk score was negatively associated with immune infiltration levels of immune infiltrates of M1 macrophages, resting mast cells, and neutrophils.

**Figure 12:**
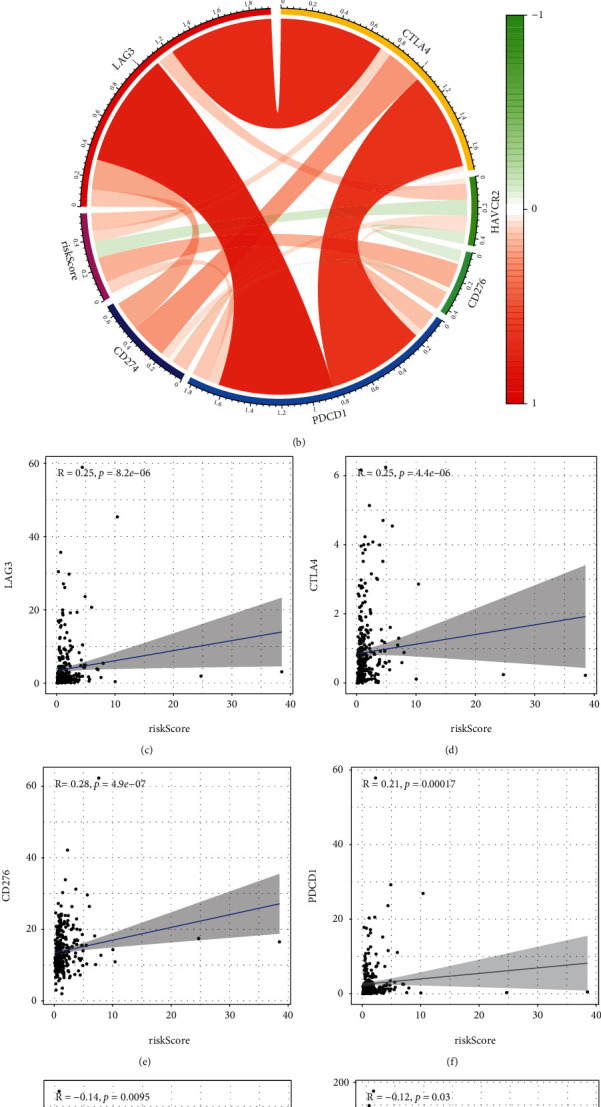
The comparison of immune checkpoint expressions between low and high subgroups. (a) Cases with high-risk score expressed increased levels of LAG-3, CTLA4, CD276, and PDCD1, whereas HAVCR2 and CD274 were highly expressed in patients with low-risk score. (b) The circus map of the correlation between risk score and LAG-3, CTLA4, HAVCR2, PDCD1, CD276, and CD274. (c–f) LAG-3, CTLA4, CD276, and PDCD1 were positively related to the risk scores, whereas (g, h) CD274 and HAVCR2 were negatively related to the risk scores.

ESTIMATE showed that immune infiltration was distinctly lower in the low-risk groups compared to the high-risk groups, which was not unexpected (*P* < 0.01; Figure 11(a)), and the stromal score did not alter in any way (Figure 11(b)). Then, we used the CIBERSORT algorithm to examine the estimated fraction of 22 immune cells. Samples with a calculated *P* value of <0.05 were included to assure the analysis's accuracy, and the results are illustrated in Figure 11(c). Pearson correlation analysis revealed that risk scores were positively related to immune infiltration levels of memory B cells (Figure 11(d)), plasma cells (Figure 11(e)), activated memory CD4+ T cells (Figure 11(f)), follicular helper T cells (Figure 11(g)), regulatory T cells (Tregs) (Figure 11(h)), and M0 macrophages (Figure 11(i)), whereas risk score was negatively associated with immune infiltration levels of immune infiltrates of M1 macrophages (Figure 11(j)), resting mast cells (Figure 11(k)), and neutrophils (Figure 11(l)). As displayed in Figure 12(a), cases with high-risk scores displayed increased expressions of PDCD1, CTLA4, LAG-3, and CD276, whereas HAVCR2 and CD274 were highly expressed in patients with low-risk score.  Figure 12(b) shows the circus map of the correlation between risk score and immunosuppressive molecules. Specifically, LAG-3 (Figure 12(c)), CTLA4 (Figure 12(d)), CD276 (Figure 12(e)), and PDCD1 (Figure 12(f)) were positively related to the risk scores, whereas CD274 (Figure 12(g)) and HAVCR2 (Figure 12(h)) were negatively related to the risk scores.

### 3.8. The Effects of BST2 Knockdown on the Proliferation of ccRCC Cells

**Figure 13:**
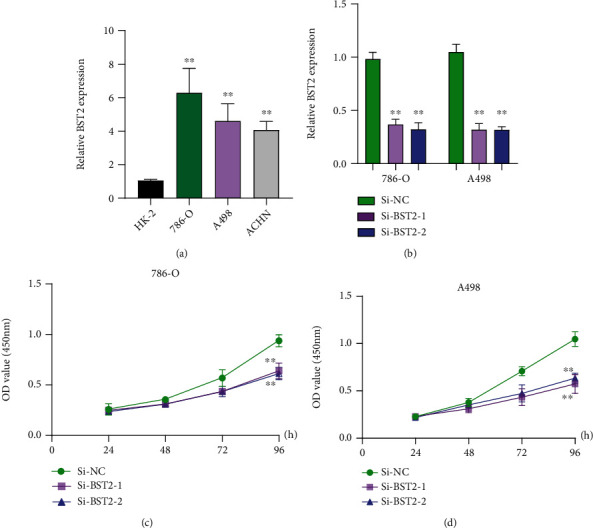
Effect of BST2 on ccRCC cells. (a) RT-PCR for the expressions of BST2 in ccRCC cells and normal HK-2 cells. (b) The relative expressions of BST2 in 786-O and A498 cells transfected with si-NC, si-BST2-1, or si-BST2-2 were examined by qRT-PCR. (c, d) Cells with transfection of si-NC, si-BST2-1, or si-BST2-2 were examined by the CCK-8 assay. ^∗∗^*P* < 0.01. Student's *t*-test was performed to analyze the significance of differences between two groups.

Then, our group detected the expressions of BST2 in ccRCC cells by the use of RT-PCR. As shown in Figure 13(a), BST2 expression was distinctly increased in three ccRCC cells compared with HK-2 cells. To study the potential functions of BST2 in ccRCC, our group constructed siRNA targeting BST2, and then, si-BST2-1 and si-BST2-2 were transfected into 786-O and A498 cells to downregulate BST2 expression (Figure 13(b)). Next, CCK-8 assays showed that BST2 knockdown suppressed the proliferation of 786-O and A498 cells (Figures 13(c) and 13(d)). Our findings suggested BST2 as an oncogene in ccRCC cells.

## 4. Discussion

Cell death is the irreversible cessation of life phenomena and the end of life; it occurs in normal specimens and is necessary to maintain organizational functions and morphologies. In tumor cells, accelerating cancer cell death can inhibit cancer progression and angiogenesis. Pyroptosis is an inflammatory form of cell death triggered by specific inflammatory vesicles, which can lead to the cleavage of GSDMD/GSDME and activation of certain inactive cytokines [11]. Pyroptosis was closely associated with certain diseases such as cardiovascular and neurological disorders [17, 18]. Recently, growing researches have found that pyroptosis played a significant role in in the occurrence, developments, and treatment of tumors. Several pyroptosis-related genes have been proved to be related to cancer progression. For instance, in pancreatic cancer, loss of MST1inhibited the progression of tumor cells at least partially through ROS-induced pyroptosis [19]. In non-small-cell lung cancer, overexpression of p53 significantly reduces tumor growth and mortality by increasing the level of pyroptosis in vivo and vitro, while loss of p53 is the opposite [20]. Tom20 senses iron and then transmitted ROS to the mitochondria, thereby inducing pyroptosis in melanoma cells [21]. Besides, certain compounds, natural substances, and drugs have proved their effects on pyroptosis. Qiao et al. [22] have confirmed that *α*-NETA induced pyroptosis of ovarian cancer cells via GSDMD. Zheng et al. [23] have revealed that metformin induced mitochondrial dysfunctions, which drives caspase-3/GSDME-mediated pyroptosis in cancer cells. Pizato et al. [24] showed that Omega-3 induced pyroptosis cell death in breast cancer cells. Hu et al. have demonstrated that disulfiram could inhibit pyroptosis by blocking the gasdermin D pore formation. Deng et al. [25] have suggested that BIX-01294 promoted chemotherapy effects in gastric cancer via modulating GSDME-mediated pyroptosis. In general, pyroptosis has attracted widespread attention, and inducing pyroptosis cancer cells offers a new approach to antitumor therapy. In a previous study, pyroptosis-related signature was identified as independent prognostic factors for esophageal adenocarcinoma and was used to assess patient's risk stratification [26]; however, the potential values of pyroptosis-associated signature for the prediction of clinical outcome in ccRCC are unclear.

In this study, we investigated the signaling pathways closely correlated with the risk scores. Herein, 11 hub PRGs (CASP9, TUBB6, NFKB1, BNIP3, CAPN1, CD14, PRDM1, BST2, SDHB, TFAM, and GSDMB) were applied to successfully construct an 11-PRG risk signature. Subsequently, we found that the risk sore performed well in all groups. This is reflected in the higher-risk scores indicating a poorer prognosis for patients in all four groups. Besides, we found that the 11-PRG genetic risk model independently predicted overall survival in ccRCC patients, and that the risk score was effective in predicting 3-year and 5-year OS of patients with ccRCC. Next, we built a predictive nomogram utilizing the risk score and other clinicopathological factors, which was also confirmed in ROC assays. Taken together, we constructed a robust 11-gene signature associated with apoptosis and built a validated nomogram for prognostic prediction in ccRCC.

In the present study, 11 hub PRGs (CASP9, TUBB6, NFKB1, BNIP3, CAPN1, CD14, PRDM1, BST2, SDHB, TFAM, and GSDMB) were included in the risk signature. They were associated with pyroptosis, some of which are associated with the progression of malignant tumors. CASP9 (caspase-9), a member of the cysteine-aspartic acid protease (caspase) family, was confirmed to play a crucial role in cancer progression [27, 28]. Tsuchiya et al. [29] found that CASP9 plays a key role in the process of caspase-1-mediated pyroptosis in GSDMD-deficient cells. Salinas et al. [30] confirmed that pharmacological reduction of TUBB6 expression or stabilization of microtubules with paclitaxel (Taxol) increases pyroptosis. NFKB1 (nuclear factor kappa B subunit 1), also known as NF-*κ*B, is a transcriptional regulator that can be activated by multiple intracellular and extracellular factors, and the activated NFKB translocates further into the nucleus to modulate the expression of genes with multiple biological functions [31]. In lung cancer, the NF-*κ*B signaling pathway was reported to be closely associated with the regulation of apoptosis in tumor cells [32]. BNIP3 (BCL2 interacting protein 3), also known as NIP3, encodes a mitochondrial protein that is a proapoptotic factor. Dysregulation of BNIP3 expression is associated with mitophagy, autophagy, and pyroptosis [33–35]. CAPN1 (calpain 1) is a member of the calpains that are nonlysosomal, intracellular cysteine proteases. A recent report indicated that CAPN1 serves as a promoter of cancer progression, and abnormal expression of CAPN1 promotes malignant behavior of various tumors [36–38]. Additionally, loss of CAPN1 reduces myocardial ischemia-reperfusion injury through the pyroptosis in mice [39]. Emerging evidence showed that CD14 is a multitalented receptor, which was distinctly involved in the pathogenesis of inflammation, atherosclerosis, tumor, and metabolic diseases [40]. It was reported that its receptor (TLR4) could mediate pyroptosis in human hepatoma-derived HuH-7 cells [41]. Chai et al. [42] revealed that abnormal PRDM1 expression is closely correlated with the proliferation and metastasis of colon cancer cells. BST2 (bone marrow stromal cell antigen 2) encodes a protein that may play a role in pre-B cell growth and in rheumatoid arthritis. SDHB is the subunit of the succinate dehydrogenase complex, which was mainly involved in the oxidation of succinate, carries electrons from FADH to CoQ. Patients with SDHB mutations are susceptible to malignant disease with limited therapeutic chooses and adverse prognosis [43]. Wu et al. [44] found that the SDHB/ROS pathway was involved in the induction of pyroptosis and promotion of atherosclerosis in mice. TFAM (mitochondrial transcription factor A) encodes a hub mitochondrial transcription factor, which mainly functions in mitochondrial DNA replication and repair. Integrated genomics analysis confirmed that TFAM is a critical driver in drug resistance in melanoma [45]. Several researches reported that GSDMB expression was distinctly increased in several types of tumors [46]. Additionally, Panganiban et al. found that cleavage of GSDMB protein by caspase-1 induces pyroptotic cell death [47]. They demonstrated the roles of 11 hub PRGs in pyroptosis as well as in carcinogenesis. However, whether CASP9, TUBB6, NFKB1, BNIP3, CAPN1, CD14, PRDM1, BST2, SDHB, TFAM, and GSDMB played a role remained to be further studied, and there are few relevant studies.

Then, we performed GO and KEGG assays and observed that the enriched biological function terms were mainly related to cell proliferation and differentiation. Interestingly, KEGG pathway results showed that DEGs are mainly involved in neuroactive ligand-receptor interaction, IL-17 signaling pathway, and calcium signaling pathway, and we therefore speculated that the pyroptosis-related risk signature might be related to tumor immunity. Accumulating evidence demonstrated that tumor microenvironment regulated tumorigenesis and progressions. In this research, our group observed that the high-risk group exhibited higher immune scores, suggesting the high-risk group showed a higher degree of immune cell infiltration compared to the low-risk group. However, they promoted tumor progression by suppressing effective antitumor immunity [48]. What is more, elevated levels of M0 macrophages have been demonstrated to be related to an unfavorable clinical prognosis of cancer [49]. A previous study suggested that LAG-3 knockdown resulted in increased T cell [50]. B7-H3 (CD276) plays a vital role in suppressing T cell function. Some researchers found that that B7-H3 was significantly overexpressed in various human solid cancers and often associated with poor clinical outcomes [51]. PDCD1 (PD-1) plays critical roles in T cell coinhibition and exhaustion, and increased levels of PD-1 predicted disease progressions in many types of tumors [52]. Immune checkpoint inhibitors may be more beneficial for high-risk individuals, according to the aforementioned studies.

## 5. Conclusions

Our research constructed and validated a robust 11-PRG risk signature. However, further in vitro and in vivo experiments with larger sample sizes must be performed to explore the exact biological roles behind genes associated with apoptosis and survival outcomes in ccRCC.

## Data Availability

The data used to support the findings of this study are available from the corresponding author upon request.

## Supplementary Materials

Supplementary 1 Table S1: univariate Cox regression analysis for identification of prognosis-related PRGs in the training dataset.

Supplementary 2 Table S2: 11 prognosis-associated hub PRGs identified by multivariate Cox regression analysis.
