# Supplementary material for: A Novel Pyroptosis-Related Prognostic Signature for Risk Stratification and Clinical Prognosis in Clear Cell Renal Cell Carcinoma
Source: Dis Markers. 2022 Mar 9;2022:8093837. doi: 10.1155/2022/8093837 (PMC8927973; doi:10.1155/2022/8093837)
Supplement: Supplementary 1 — Table S1: univariate Cox regression analysis for identification of prognosis-related PRGs in the training dataset. [file 8093837.f1.pdf]

Table S1. Univariate Cox regression analysis for identification of prognosis-related PRGs in the training dataset.

ID	HR	HR.95L	HR.95H	p-value
LRPPRC	0.408	0.285	0.584	9.470E-07
SDHB	0.297	0.180	0.489	1.890E-06
TXNIP	0.583	0.457	0.743	1.290E-05
IRF3	2.432	1.631	3.628	1.320E-05
CEBPB	1.538	1.267	1.867	1.350E-05
GSDMB	1.831	1.394	2.405	1.390E-05
DHX9	0.441	0.305	0.639	1.460E-05
NFKB1	0.478	0.337	0.676	3.140E-05
TFAM	0.369	0.230	0.592	3.460E-05
PECAM1	0.708	0.595	0.841	8.820E-05
BCL2	0.663	0.537	0.819	1.401E-04
PYCARD	1.493	1.197	1.861	3.767E-04
BNIP3	0.675	0.542	0.841	4.469E-04
BECN1	0.500	0.339	0.737	4.649E-04
AGER	1.490	1.191	1.863	4.770E-04
CASP9	2.530	1.486	4.310	6.335E-04
GJA1	0.746	0.624	0.893	1.368E-03
ATF6	0.507	0.333	0.771	1.508E-03
HUWE1	0.584	0.419	0.815	1.548E-03
CASP4	2.053	1.314	3.206	1.575E-03
AIM2	1.425	1.136	1.788	2.170E-03
IFI16	1.681	1.201	2.352	2.438E-03
IRGM	29.534	3.041	286.850	3.514E-03
ZBP1	1.619	1.171	2.238	3.553E-03
CASP5	2.310	1.312	4.068	3.729E-03
GPER1	0.764	0.635	0.918	4.012E-03
EGFR	0.723	0.571	0.915	6.927E-03
NFE2L2	0.526	0.330	0.839	7.031E-03
CASP3	1.956	1.177	3.249	9.611E-03
NR1H2	2.206	1.187	4.100	1.233E-02
NLRP1	1.471	1.081	2.000	1.399E-02
MEFV	1.958	1.143	3.354	1.447E-02
GLMN	2.250	1.155	4.382	1.717E-02
LY96	1.291	1.044	1.595	1.826E-02
ANO6	0.681	0.491	0.944	2.097E-02
IL13	32.368	1.598	655.686	2.350E-02
TUBB6	1.491	1.047	2.124	2.692E-02
GSDMD	1.566	1.051	2.335	2.768E-02

APOL1	1.176	1.016	1.362	3.014E-02
CAPN1	0.548	0.317	0.949	3.173E-02
GSTO1	1.389	1.028	1.877	3.250E-02
BST2	1.300	1.021	1.656	3.358E-02
ANXA2	1.391	1.024	1.889	3.466E-02
PRDM1	0.750	0.572	0.983	3.685E-02
NEK7	0.676	0.466	0.981	3.924E-02
CD14	1.262	1.006	1.582	4.433E-02
