# Supplementary material for: A Novel Pyroptosis-Related Prognostic Signature for Risk Stratification and Clinical Prognosis in Clear Cell Renal Cell Carcinoma
Source: Dis Markers. 2022 Mar 9;2022:8093837. doi: 10.1155/2022/8093837 (PMC8927973; doi:10.1155/2022/8093837)
Supplement: Supplementary 2 — Table S2: 11 prognosis-associated hub PRGs identified by multivariate Cox regression analysis. [file 8093837.f2.pdf]

Table S2. 11 prognosis-associated hub PRGs identified by multivariate Cox regression analysis.

ID	coef	HR	HR.95L	HR.95H	pvalue
CASP9	1.174	3.236	1.659	6.315	5.74E-04
TUBB6	0.359	1.432	1.001	2.049	4.97E-02
NFKB1	-0.6	0.549	0.304	0.99	4.63E-02
BNIP3	-0.404	0.667	0.507	0.879	4.03E-03
CAPN1	-0.827	0.438	0.233	0.82	9.94E-03
CD14	0.404	1.498	1.175	1.909	1.10E-03
PRDM1	-0.643	0.526	0.373	0.741	2.37E-04
BST2	0.225	1.252	1	1.567	4.96E-02
SDHB	-0.544	0.581	0.315	1.07	8.14E-02
TFAM	0.973	2.647	1.199	5.843	1.60E-02
GSDMB	0.307	1.359	0.972	1.902	7.32E-02
